# Enhancing Focus and Short Reaction Time in Épée Fencing: The Power of the Science Vision Training Academy System

**DOI:** 10.3390/jfmk9040213

**Published:** 2024-10-30

**Authors:** Giulia Di Martino, Stefano Giommoni, Fosco Esposito, Davide Alessandro, Carlo della Valle, Enzo Iuliano, Giovanni Fiorilli, Giuseppe Calcagno, Alessandra di Cagno

**Affiliations:** 1 Department of Medicine and Health Sciences, University of Molise, 86100 Campobasso, Italy; giulia.dimartino21@gmail.com (G.D.M.); fiorilli@unimol.it (G.F.); giuseppe.calcagno@unimol.it (G.C.); 2 Italian Fencing Federation, Viale Tiziano 74, 00196 Rome, Italy; stefanogiommoni@gmail.com (S.G.); espopiana@gmail.com (F.E.); alessandro.davide@libero.it (D.A.); 3 Department of Neurosciences, Biomedicine and Movement, University of Verona, 37134 Verona, Italy; 4 Faculty of Psychology, eCampus University, 22060 Novedrate, Italy; 5 Faculty of Medicine, University of Ostrava, 73000 Ostrava, Czech Republic; 6 Department of Movement, Human and Health Sciences, University of Rome “Foro Italico”, 00135 Rome, Italy; alessandra.dicagno@uniroma4.it; 7 Department of Human Sciences, Guglielmo Marconi University, Via Plinio 44, 00193 Rome, Italy

**Keywords:** visual training, inhibition, executive functions, fencers

## Abstract

**Background:** This study aimed to evaluate the effects of a six-week visual training protocol, based on the Science Vision Training Academy (SVTA) method, on reaction times and executive functions in high-ranking fencers. **Methods:** Twenty-seven fencers, aged 17.34 ± 3.63 years, were randomly assigned to an experimental Visual Training Group (VTG = 16) and a Control Group (CG = 11). The VTG, in addition to regular fencing training, underwent SVTA training two times per week using six different visual modules, while the CG followed only their traditional fencing training. Simple and complex reaction times and movement times were assessed before and after the intervention using the Fit-Light System. **Results:** Both groups showed a significant improvement in all four reaction time tests: simple reaction time with and without a weapon and complex reaction time ability (motor inhibition ability) with and without a weapon (*p* < 0.001). No significant differences were observed between the groups. A significant Time* Group interaction was found in the short reaction time and movement time (*p* < 0.001). This trend suggests that, although genetically determined and difficult to significantly improve through training, short reaction time can be stimulated through SVTA protocols. **Conclusions:** Training in realistic conditions is always preferable to non-ecological protocols; however, the SVTA method may be beneficial to enhance simple reaction time in elite fencers.

## 1. Introduction

Fencing is a combat sport that requires very fast attack and defense movements under pressure: in fencing, athletes need to analyze opponents’ assaults, interpret the action intentions of their opponents, and have a fast reaction time. Optimal anticipation and concentration due to the proficiency of athletes in visual variables, such as accuracy and visual quality, are associated with their fencing performance as the athletes need to follow the movement of the bodies and arms of the opponents [1].

Perception and attention are distinct processes, but they are often interrelated. Attention provides a foundational basis for perception and typically occurs first; however, perception can interfere with attentional processes [2].

“Sports Visual Training” (SVT) is a vision training intervention that provides participants with intermittent visual feedback or information while they perform tasks requiring high temporal and spatial precision. The benefits include improvement in reaction time, attention, visual function, and sport-specific visuomotor performance [3]. Based on practicing demand, this training leads to faster sensory processing, quicker and more accurate motor movements, and, consequently, improved athletic performance [4]. Athletes achieve higher results when they demonstrate more efficient eye movements and, consequently, a short reaction time (SRT), better perceptual skills due to better detection, and higher-level performance in processing speed and attention measures [5].

By SRT, we mean the interval between the appearance of the stimulus and the end of the muscular response activity. The speed of a simpler response is determined by the efficiency of the nervous system’s information processing rate and is individually genetically determined [6].

The requirement for a more complex response involving a precise motor task depends more on the athlete’s experience and conditioning level and is referred to as movement time (MT) [7,8]. Therefore, we refer to MT as the time required for the athlete to complete a fencing attack.

Sports training significantly reduces MT. Moreover, elite athletes select the most important stimuli, which are keys for a given action [9]. Nevertheless, an SRT is crucial in achieving excellent fencing performance [7].

SVT could be useful to enhance fencers’ attentional and visual skills. Considering that athletes heavily rely on their visual and proprioceptive systems for precise movements and actions, assessing the integration between the visual and motor systems is crucial, highlighting the need to train and enhance visuomotor skills [10].

A key benefit of SVT compared to other visual training methods, whether paper- or computer-based, is that it allows training to be carried out in practical real-world environments.

The authors of the present study designed a specific training protocol for fencers based on the Science Vision Training Academy (SVTA) method and materials, which integrate the visual, cognitive, and motor systems [11]. The SVTA method uses a work kit consisting of different-sized charts to execute a visuomotor training program for enhancing specific visual skills. On one hand, this method focuses on improving hand–eye coordination by reducing latency times between stimulus processing and the execution of technical motor actions; on the other hand, this method enhances the readiness of a response to a specific stimulus, thereby making the selection process faster (selection stage). SVTA involves different skills, including visual skills, the ocular vestibular system, kinesthetic sense, coordinative motor skills, balance, and cognitive abilities [12].

This study aimed to assess improvements in reaction time and executive functions following six weeks of training based on SVTA methods. The response to the visual stimulus required behaviors, without and with a weapon, to be performed under realistic sports situations (e.g., extended arm, lunge, step back, and step forward). The assessment was also designed to be “ecological”, and responses to visual stimuli, produced by the Fit-Light System, were evaluated both with and without a weapon. To isolate the effects of the SVTA training protocol, the final results were compared with those of athletes from the same weapon category and skill level who followed traditional fencing training, considered as a control group.

## 2. Materials and Methods

### 2.1. Study Design

The present study is a Randomized Controlled Trial designed to evaluate the effects of 6 weeks of a specific SVTA training protocol, adapted for fencers’ performance, compared to those of traditional training. Twenty-seven fencers were randomly assigned to the experimental Visual Training Group (VTG = 16) or the Control Group (CG = 11). The VTG trained with specific technical movements using six different visual training modules to assess the effectiveness in enhancing reaction time and executive functions.

### 2.2. Participants

This study involved a population of 31 epee fencers with a high level ranking, from the Cadet and Junior categories, of both genders (man = 16; female = 15) aged 17.34 ± 3.63 years. In the preliminary phase, each participant was assigned to a sequential number. Subsequently, a list of random numbers was generated through online software (https://www.random.org/sequences/ (accessed on 4 September 2023), Dublin, Ireland), and the subjects were assigned to the VTG or CG. During the intervention period, there were four dropouts in the control group due to injuries, and two athletes changed clubs, reducing the final total of participants to 27 epee fencers (VTG = 16 and CG = 15). Since the protocol had already started, further randomization of the sample was not possible. Since previous studies have not found significant differences between genders in SRT, visual perception, and perceptual-motor skills [1,13], the sample was considered as a single group.

The inclusion criteria were (a) an age between 15 and 20 years; (b) a training frequency of at least 4 sessions per week; and (c) a rank within the top 50 positions in their category. The exclusion criteria were (a) injuries that occurred in the previous 3 months; (b) the use of drugs or medications or other conditions that could influence the test results; and (c) having already undergone training using the SVTA method.

All participants were informed about the objective and procedures of this study and signed an informed consent form. For under-aged participants, informed consent was provided by their parents or guardians. This study was designed and conducted by the Declaration of Helsinki and approved by the Bioethical Local Committee of University of Rome “Foro Italico” (University Committee for Research—CAR-IRB—Code: CAR 15/2023).

### 2.3. Procedures

#### 2.3.1. Assessment

At baseline, all the participants underwent a testing session, which was repeated after 6 weeks at the end of the protocol.

Data collection was carried out using the Fit-Light System™ (Fitlight Corp, Aurora, ON, Canada, 2011) (which included results for the average SRT and MT execution) [14]. The Fit-Light System consisted of 5 disks with LED lights and a central wireless controller that recorded the test results, providing accurate real-time data that were accessible and could be downloaded through standard laptop applications.

The five lights were positioned at different heights and distances for all tests, illuminating intermittently for a predetermined duration according to a sequence unknown to the athlete. Each test was performed with three measurements, and the best one was considered for the analysis. The tests, along with their respective measurements of the SRT and MT, were conducted as follows:

(a) Test 1: *Simple Reaction Time (SRT)*. During the test, one light was turned on at a time with an interval of 10 s between lights. Extending their dominant arm, the athlete had to turn off 50 lights by touching them. The average SRT and the MT were calculated.
(b) Test 2: *Simple Reaction Time with a Weapon (SRTW)*. During the test, one light was turned on at a time with an interval of 10 s between lights. The athlete, in the guard position, had to turn off 50 lights by touching them with a weapon. The average SRT and the MT were calculated.
(c) Test 3: *Complex Reaction Ability (motor inhibition ability)*. Multiple lights of three different colors (red, blue, and green) turned on simultaneously. The athlete had to respond to different visual stimuli, select the correct one, and hit only the blue lights, ignoring the other stimuli. Extending their dominant arm, the athlete had to turn off 25 lights by touching them. The MT, average SRT, and number of errors were calculated.
(d) Test 4: *Complex Reaction Ability with a Weapon*. Following the same procedure as the complex reaction ability test, the athlete, in the guard position, had to turn off 25 blue lights by touching them with a weapon. The MT, average SRT, and number of errors were calculated.

#### 2.3.2. Visual Training Charts

The intervention protocol involved visual training exercises using six different main charts, which are square-shaped with standard dimensions of 50 × 50 cm. To maintain the correct posture required for executing specific fencing movements (such as step forward, step back, arm extension, and lunge) and maximum uniformity during the test, a standard height was defined. This height ranges from 140 to 160 cm from the floor, measured from the top edge of the charts. In addition to the main charts, supplementary charts (minor charts) with similar symbols, measuring 25 × 25 cm, may be positioned laterally. Each chart included exercises with four different levels of difficulty of coordinative and attentive tasks. A standardized execution order was established for all participants. Detailed explanations of the six charts are provided in the Supplementary Materials (Table S1 and Figure S1).

#### 2.3.3. Protocol Intervention

**Figure 1:**
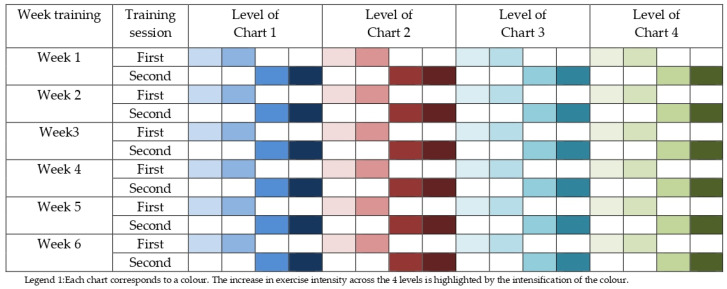
Organization of six-week experimental training program.

The study protocol was performed two times per week after the fencing training session. All participants performed the same traditional fencing training session during the intervention period. During each session, three out of six charts were used and positioned at separate stations (Figure 1). Participants performed 2 min of timed work at each station, resulting in a total of 12 min of training per day. The exercises on each chart varied in difficulty in each session (four levels). Charts were read starting from the top row and proceeding from left to right. Participants were required to restart the exercise from the top row, without stopping the timer, if an error occurred.

### 2.4. Statistical Analysis

Data are shown as means ± standard deviation (SD). The Shapiro–Wilk test was used to assess the normality of continuous variables. A one-way Analysis of Variance (ANOVA) was then employed to determine if the two groups (VTG vs. CG) were homogenous in terms of age, years of experience, and baseline reaction time test scores, which are all considered continuous variables. The Chi-Square analysis (*χ*^2^) was used to evaluate the homogeneity of the two groups in terms of gender distribution, which was considered a categorical variable.

A repeated-measures multivariate ANOVA (RM-MANOVA) between factors was performed to assess significant differences within the pre- vs. post-intervention reaction time test scores (within a factor of the analysis named Time), between the two groups (between factors of the analysis named Group), and in the Time* Group interaction. SRT and MT for each reaction time test were considered dependent variables. Partial eta squared (*η^2^_p_*) was calculated as an estimate of the effect size, with the following criteria for interpretation: *η^2^_p_* ≥ 0.01 for a small effect, *η^2^_p_* ≥ 0.06 for a medium effect, and *η^2^_p_* ≥ 0.14 for a large effect.

For all analyses, the significance level was set at *p* < 0.05. SPSS statistical software (IBM, v.29.0, Chicago, IL, USA) was used.

## 3. Results

The Shapiro–Wilk test showed a normal distribution of the continuous variables. The one-way ANOVA showed that the two groups were homogenous in terms of age, years of experience, and reaction time test scores at baseline, while *χ*^2^ showed no significant difference in gender distribution.

The RM-MANOVA showed significant differences in the pre- vs. post-intervention reaction time test scores (F_8,18_ = 32.015; *p* < 0.001; *η*^2^*_p_* = 0.934). A significant difference was found between the two groups (F_8,18_ = 2.540; *p* = 0.048; *η*^2^*_p_* = 0.530) and in the Time* Group interaction (F_8,18_ = 3.387; *p* = 0.015; *η*^2^*_p_* = 0.601). The univariate analysis showed a significant improvement in the SRT scores in all four reaction time tests (all with *p* < 0.001), with no significant change in the MT score in any of the four tests. The univariate analysis did not confirm the differences between groups. The analysis performed on each one of the dependent variables separately showed no differences between the VTG and CG both in the MT or SRT scores on the four tests. Finally, the univariate analysis showed a significant Time* Group interaction in the SRT and MT of Test 1 (respectively, with F_1,25_ = 9.449, *p* = 0.005, and *η*^2^*_p_* = 0.274 and F_1,25_ = 12.713, *p* = 0.001, and *η*^2^*_p_* = 0.337), with better results being obtained for the VTG. In Table 1, the complete results are reported.

## 4. Discussion

The training protocol chosen for this study was designed to enhance the ability to generate responses to a given stimulus under both compatible and incompatible conditions. Additionally, it aimed to enhance the ability to inhibit instinctive responses to stimuli and improve efficient responses to a given stimulus [13].

Tests 1 and 2 were designed to evaluate the simple reaction time without and with a weapon, respectively. Tests 3 and 4 assessed the complex reaction time with inhibition and without and with a weapon. Both groups of high-ranking-level athletes, who underwent the same fencing training, showed a significant improvement over the 6-week intervention period both in the SRT and MT. Fencing training develops ecological (specific) solutions, enhancing the athlete’s ability to decide which stimulus (visual–tactile) to attend to and focus on (narrow focus) while simultaneously improving the athlete’s ability to maintain awareness of environmental stimuli (broad focus) in which the attack occurs [12]; consequently, no differences between the groups were found in either the reaction time (SRT) or movement time (MT).

The results that we obtained were related more to improvements in simple reactions rather than complex reactions, which are more effectively stimulated by traditional fencing training. The ability to inhibit responses is more effectively stimulated by real-world situations than by the SVTA protocol. Nevertheless, the significant Time* Group interaction, observed for the short reaction time and relative movement time, after the SVTA method, is an important finding as the scientific literature suggests that simple reactions are generally less trainable than complex ones [1].

Fencing training, due to its nature, stimulates both visual acuity and task focus [12], and athletes recognize stimuli, particularly those specific to the task, for initiating a response more quickly [13]. Moreover, fencing training not only stimulates this initial phase of information processing but also enhances the speed of organizing the complex motor responses.

Regarding exercises three and four, the athletes had to inhibit their instinctive response to perform the required one. The improvement in inhibition action is a result of an intense conflict of control developed by fencers after stimulus response alternatives [13]. Several studies assessed that physical training significantly modulated executive functioning, particularly inhibition, especially in acute conditions [15,16]. In performing the third and fourth exercises, participants train their inhibition ability by accurately reading the protocol cards and constantly changing their motor responses. However, the ability to inhibit responses was found to be more effectively stimulated by real-world situations than by the SVTA protocol. The ecological context of fencing performance enhances the athlete’s capacity to concentrate, select the relevant stimulus, and improve their focus and attention in response to the opponent’s actions. Attention allows perception to occur, and fencing training simultaneously stimulates attention, concentration, and perception [1].

For these reasons, it was decided to compare the two groups of athletes (VTG and CG) at the same technical level, performing the same usual training, to isolate the effects of the SVTA protocol. No significant differences were found between the two groups of fencers. This suggests that the fencers’ ability to respond to an opponent’s feint and quickly switch from an intended action to a more appropriate one is likely more effective than the SVTA protocol in inhibition action. The higher complexity of attack initiation in realistic situations likely enhanced the inhibition action from a coordinative standpoint.

The significant interaction between the groups in the first exercise, favoring the group who underwent the SVTA protocol (the VTG), showed a trend of better improvement in the SRT following the visual training protocol. This result was related to a trend of improvements in the simple reaction time rather than complex reactions. This finding is significant because the scientific literature highlights that complex reactions are much more trainable than simple ones [1].

The simple reaction time, where a subject responds to a visual stimulus with a basic movement, is highly influenced by genetics and less affected by training [9]. In fact, previous studies have shown that there are no significant differences in the SRT between beginners and the sports-active population [16,17]. The SRT is a key factor in fencing as it is closely related to decision making [18]. A positive correlation between a fast SRT and a lower percentage of errors in combat was previously demonstrated [19]. Furthermore, SRT training enhances levels of focused attention (narrow focus), which are particularly beneficial in fencing. Considering that attacks in fencing last only fractions of a second, visual perception and SRT become crucial components of performance and should be emphasized in training plans, especially for elite athletes [1]. The SVTA training task focuses on isolating and developing this specific ability, which typically interacts with other skills through a complex perceptual process [20], thereby accelerating athletes’ visuomotor reactions [19]. Neurophysiological experiments have shown that a faster visuomotor reaction is due to a better ability to process visual information [3]. Moreover, the retention of visual information is strongly correlated with the ability to maintain a stable position and assume the target position to effectively counter the opponent [2,21].

These results suggest that the SVTA method may have potential effects to improve the SRT that are crucial for fencing performance and challenging to improve [22,23]. It is important to note that the sample analyzed consists of elite athletes, for whom performance improvements are generally more challenging due to the “ceiling effect” associated with long-term training [24,25]. Discovering an additional tool that enhances SRT is especially important in a sport like fencing, where quick reaction times are crucial for success [26]. This protocol, which enhances SRT, differs from the usual training program and could be used during the transition period away from competitions. It contributes to the variation in methods, a crucial aspect of an effective training program.

A few potential limitations should be acknowledged and taken into consideration for future research.

The results of this study are applicable to elite athletes aged 17 to 20 years and therefore cannot be generalized to athletes of different technical levels or ages. Further studies could apply the SVTA protocol to other age groups or different skill levels.

Based on the assumption that male and female athletes do not significantly differ in reaction times [1], the sample was not divided by gender.

## 5. Conclusions

In conclusion, fencing training and competitions significantly stimulate the complex reaction time, both in terms of the speed of offensive and defensive actions, by familiarizing athletes with these stimuli. Significant results did not emerge because the control group continued to train in fencing performance, where visual acuity and attention are still engaged, often in conjunction with other skills, thus providing specificity to training. In contrast, with SVTA, visual acuity and SRT are trained in isolation with the goal of improving a skill that is difficult to train.

Moreover, it is crucial to explore innovative methodologies that can further enhance performance, especially for elite athletes. Experience certainly improves the effectiveness of their actions, particularly in terms of extracting multiple inputs simultaneously, processing them concurrently, and directing attention to what is important at the moment. However, improving SRT also influences the speed of more complex aspects of performance.

While SRT is genetically determined and difficult to improve significantly through training, it can be stimulated through SVTA protocols. The benefit of implementing the training protocol with new tools is evident not only in the improvement trend observed within the VTG but also in the contribution to method variation, which is a crucial factor in enhancing performance and designing an effective training program.

Training under realistic conditions is always preferable to non-ecological protocols. However, this method can be beneficial to improve SRT, particularly during periods away from the competitive season.

## Figures and Tables

**Table 1:** The results of the VTG and CG in the four reaction time tests.

Test	Test (SRT and MT)	VTG		CG		*p*-Value VTG vs. CG	*p*-Value Pre vs. Post	*p*-Value Time * Groups
		Pre	Post	Pre	Post			
Simple Reaction Time	SRT-T1	0.43 ± 0.04	0.39 ± 0.03 *	0.43 ± 0.03	0.42 ± 0.04	*p* = 0.225 (F= 1.551)	*p* < 0.001 ^#^ (F = 16.901)	*p* = 0.005 ^#^ (F = 9.449)
	MT-T1	25.5 ± 2.22	24.51 ± 1.42 *	25.27 ± 1.65	25.78 ± 2.25	*p* = 0.476 (F= 0.523)	*p* = 0.262 (F = 1.316)	*p* = 0.001 ^#^ (F = 12.713)
Simple Reaction Time with a Weapon	SRT-T2	0.46 ± 0.04	0.43 ± 0.03	0.48 ± 0.03	0.46 ± 0.04	*p* = 0.091 (F = 3.085)	*p* < 0.001 ^#^ (F = 16.341)	*p* = 0.386 (F = 0.779)
	MT-T2	27.25 ± 2.11	26.26 ± 1.67	27.79 ± 2.00	27.98 ± 2.41	*p* = 0.129 (F= 2.468)	*p* = 0.264 (F = 1.303)	*p* = 0.102 (F = 2.883)
Complex Reaction Ability	SRT-T3	0.64 ± 0.06	0.58 ± 0.06	0.62 ± 0.04	0.58 ± 0.04	*p* = 0.450 (F = 0.588)	*p* < 0.001 ^#^ (F = 28.096)	*p* = 0.315 (F = 1.051)
	MT-T3	27.86 ± 1.28	27.42 ± 0.79	27.23 ± 0.77	27.25 ± 0.66	*p* = 0.207 (F = 1.680)	*p* = 0.301 (F = 1.115)	*p* = 0.272 (F = 1.263)
Complex Reaction Ability with a Weapon	SRT-T4	0.58 ± 0.06	0.53 ± 0.05	0.56 ± 0.05	0.54 ± 0.06	*p* = 0.834 (F = 0.045)	*p* < 0.001 ^#^ (F = 18.066)	*p* = 0.189 (F = 1.826)
	MT-T4	26.49 ± 0.83	26.27 ± 0.67	26.21 ± 0.75	26.28 ± 0.34	*p* = 0.588 (F = 0.301)	*p* = 0.532 (F = 0.402)	*p* = 0.242 (F = 1.437)

SRT = Short Reaction Time; MT = Movement Time; T1, T2, T3, and T4 = Test 1, Test 2, Test 3, and Test 4. * Statistically significant in comparison with pre score when 2 groups are analyzed separately due to significant Time* Group interaction. ^#^ Statistically significant in main analysis (univariate analysis).

## Data Availability

Data are available upon request to the first author: due to ethical restriction.

## Supplementary Materials

The following supporting information can be downloaded at https://www.mdpi.com/article/10.3390/jfmk9040213/s1, Table S1: Description of visual training chars. Figure S1: Illustration of the six charts used during the protocol.
