# Supplementary material for: Enhancing Focus and Short Reaction Time in Épée Fencing: The Power of the Science Vision Training Academy System
Source: J Funct Morphol Kinesiol. 2024 Oct 30;9(4):213. doi: 10.3390/jfmk9040213 (PMC11587100; doi:10.3390/jfmk9040213)
Supplement: Supplementary file 1 [file jfmk-09-00213-s001.zip › Table S1.pdf]

Table S1: Description of Visual Training Chars

Charts	*Charts utilised*	*Athletes starter position*	*Level 1*	*Level 2*	*Level 3*	*Level 4*
Chart One: red and blue mask	Main and minor charts (positioned on the sides of the main chart)	Starting from the guard position with a weapon	*Fencing movement used:* extension of the armed arm<br><br>*Type of target to hit on minor charts:* The figures should be the same colour of the mask shown in the main chart.	*Fencing movement used:* extension of the armed arm<br><br>*Type of target to hit on minor charts:* The figures should be in the colour opposite to that shown in the main chart.	*Fencing movement used:* short fencing lunge<br><br>*Type of target to hit on minor charts:* The figures should match the colour of the mask shown in the main chart.	*Fencing movement used:* short fencing lunge<br><br>*Type of target to hit on minor charts:* The figures should be in the colour opposite to that shown in the main chart.
Chart Two: Honeycomb with numbers			*Fencing movement used:* brief steps forward and step back<br><br>*Assignment:*<br>Clearly state the number first and then the same colour of the hexagon aloud.	*Fencing movement used:* brief steps forward and step back<br><br>*Assignment:*<br>Clearly state the number first and then the opposite colour of the hexagon aloud.	*Fencing movement used:* extension of the armed arm<br><br>*Assignment and Type of target to hit on minor charts:*<br>Read the number aloud, then strike the hexagon figures of the same colour.	*Fencing movement used:* extension of the armed arm<br><br>*Assignment and Type of target to hit on minor charts:*<br>Read the number aloud, then strike the hexagon figures of the opposite colour.
Chart Three: In & out			*Fencing movement used:* brief steps forward and step back<br><br>*Assignment:*<br>Perform a step either forward ("IN") or backward ("OUT") according to the instructions indicated in the cells of the main chart.	*Fencing movement used:* brief steps forward and step back<br><br>*Assignment:*<br>Perform a brief fencing step either forward ("OUT") or backward ("IN") according to the instructions indicated in the cells of the main chart.	*Fencing movement used:* brief steps forward, step back and lunge<br><br>*Assignment and Type of target to hit on minor charts:*<br>"IN" cells: first perform a step forward, then a step back, and subsequently execute a lunge to hit the same label (IN).	*Fencing movement used:* brief steps forward, step back and lunge<br><br>*Assignment and Type of target to hit on minor charts:*<br>"IN" cells: first perform a step back, then a step forward, and subsequently execute a lunge to hit the opposite label (OUT).

					"OUT" cells: first perform a step back, then a step forward, and subsequently execute a lunge to hit the same label (OUT).	"OUT" cells: first perform a step forward, then a step back, and subsequently execute a lunge to hit the opposite label (IN).
Chart Four: Masks with arrows			*Fencing movement used:* extension of the armed arm<br><br>*Type of target to hit on minor charts:* The figures should be the same direction as the arrow shown in the main chart.	*Fencing movement used:* extension of the armed arm<br><br>*Type of target to hit on minor charts:* The figures should be the opposite direction as the arrow shown in the main chart.	*Fencing movement used:* short fencing lunge<br><br>*Type of target to hit on minor charts:* The figures should be the same direction as the arrow shown in the main chart.	*Fencing movement used:* short fencing lunge<br><br>*Type of target to hit on minor charts:* The figures should be the opposite direction as the arrow shown in the main chart.
Chart Five: Numbered masks			*Fencing movement used:* short fencing lunge<br><br>*Minor charts position:* Random numerical order<br><br>*Type of target to hit on minor charts:* The figures should be the same number shown in the main chart.	*Fencing movement used:* short fencing lunge<br><br>*Minor charts position:* Random numerical order<br><br>*Assignment and Type of target to hit on minor charts:* Read the shown number, detract one, and hit the resulting number (if the result was 1, hit the number 1 anyway)	*Fencing movement used:* short fencing lunge<br><br>*Minor charts position:* Random numerical order<br><br>*Assignment and Type of target to hit on minor charts:* Read the shown number, add one, and hit the resulting number (if the result was 4, hit the number 4 anyway)	*Fencing movement used:* short fencing lunge<br><br>*Minor charts position:* Random numerical order<br><br>*Assignment and Type of target to hit on minor charts:* Read and hit the shown number. Then, hit the numbers in ascending order up to 4 (e.i. if 2 is shown, strike 2, 3, and 4 in that order). Only if the showed number is 4 should the task be performed in descending order (4, 3, 2, 1).
Chart Six: Colour chart			*Fencing movement used:* Each colour corresponds to a different task:<br><br>Red: passata-sotto	*Fencing movement used:* Each colour corresponds to a different task:<br><br>Red: passata-sotto	*Fencing movement used:* Each colour corresponds to a different task:<br><br>Red: passata-sotto	*Fencing movement used:* Each colour corresponds to a different task:<br><br>Red: passata-sotto

			Yellow: Step forward and step back.<br>Blue: Step back and step forward.<br>Green: Lunge.<br><br>*Assignment:*<br>Perform the required fencing movements, taking into account the colour "written" in the box and not the “background” colour.	Yellow: Step forward and step back.<br>Blue: Step back and step forward.<br>Green: Lunge.<br><br>*Assignment:*<br>Perform the required fencing movements, taking into account the "background" colour of the box and not the colour “written”.	Yellow: Step forward and step back.<br>Blue: Step back and step forward.<br>Green: Lunge.<br><br>*Assignment:*<br>Perform the required fencing movements, first considering the "written" colour in the box and then the “background” colour.	Yellow: Step forward and step back.<br>Blue: Step back and step forward.<br>Green: Lunge.<br><br>*Assignment:*<br>Perform the required fencing movements, first considering the “background” colour in the box and then the "written" colour.
