# Supplementary figures and images for: Enhancing Focus and Short Reaction Time in Épée Fencing: The Power of the Science Vision Training Academy System
Source: J Funct Morphol Kinesiol. 2024 Oct 30;9(4):213. doi: 10.3390/jfmk9040213 (PMC11587100; doi:10.3390/jfmk9040213)

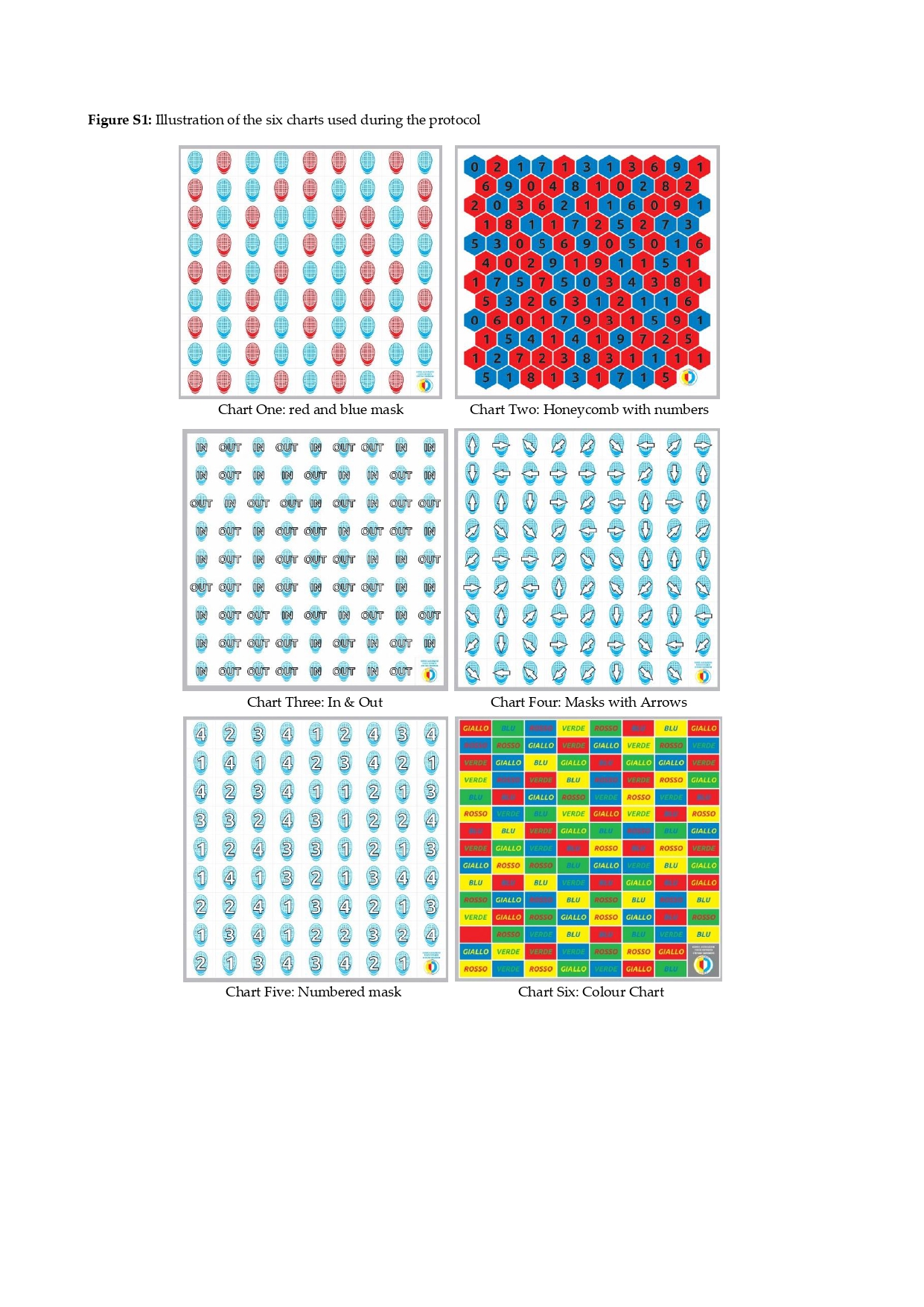

Supplement: Supplementary file 1 [file jfmk-09-00213-s001.zip › Figure S1.jpg]
